# Chemopreventive Effect of PSP Through Targeting of Prostate Cancer Stem Cell-Like Population

**DOI:** 10.1371/journal.pone.0019804

**Published:** 2011-05-16

**Authors:** Sze-Ue Luk, Terence Kin-Wah Lee, Ji Liu, Davy Tak-Wing Lee, Yung-Tuen Chiu, Stephanie Ma, Irene Oi-Lin Ng, Yong-Chuan Wong, Franky Leung Chan, Ming-Tat Ling

**Affiliations:** 1 Australian Prostate Cancer Research Centre-Queensland and Institute of Health and Biomedical Innovation, Queensland University of Technology, Brisbane, Queensland, Australia; 2 Department of Anatomy, The University of Hong Kong, Hong Kong, Hong Kong SAR; 3 Department of Pathology, The University of Hong Kong, Hong Kong, Hong Kong SAR; 4 School of Biomedical Sciences, The Chinese University of Hong Kong, Hong Kong, Hong Kong SAR; Tsan Yuk Hospital, Hospital Authority, China

## Abstract

Recent evidence suggested that prostate cancer stem/progenitor cells (CSC) are responsible for cancer initiation as well as disease progression. Unfortunately, conventional therapies are only effective in targeting the more differentiated cancer cells and spare the CSCs. Here, we report that PSP, an active component extracted from the mushroom Turkey tail (also known as *Coriolus versicolor*), is effective in targeting prostate CSCs. We found that treatment of the prostate cancer cell line PC-3 with PSP led to the down-regulation of CSC markers (CD133 and CD44) in a time and dose-dependent manner. Meanwhile, PSP treatment not only suppressed the ability of PC-3 cells to form prostaspheres under non-adherent culture conditions, but also inhibited their tumorigenicity *in vivo*, further proving that PSP can suppress prostate CSC properties. To investigate if the anti-CSC effect of PSP may lead to prostate cancer chemoprevention, transgenic mice (TgMAP) that spontaneously develop prostate tumors were orally fed with PSP for 20 weeks. Whereas 100% of the mice that fed with water only developed prostate tumors at the end of experiment, no tumors could be found in any of the mice fed with PSP, suggesting that PSP treatment can completely inhibit prostate tumor formation. Our results not only demonstrated the intriguing anti-CSC effect of PSP, but also revealed, for the first time, the surprising chemopreventive property of oral PSP consumption against prostate cancer.

## Introduction

Prostate cancer (PCa) is the most common male malignancy in western countries and represents a major disease burden in the world. When diagnosed at an advanced stage where surgery is no longer feasible, the only frontline treatment available is hormone ablation therapy. Unfortunately, the majority of PCa patients eventually relapse and develop hormone refractory PCa (HRPC), a fatal and terminal stage regarded as incurable [1].

Chemoprevention is an ideal strategy for battling prostate cancer, and a number of chemotherapeutic agents or natural food supplements are currently being tested for their potential of inhibiting prostate cancer development. For example, finasteride, a 5-alpha reductase specific inhibitor, has been shown to reduce prostate cancer incidence by 25% in a clinical trial [2]. Similarly, dutasteride, an analog of finasteride, was also reported to significantly inhibit prostate cancer development [3]. Despite of the promising result, the side-effects associated with the finasteride treatment remains the major concern for it to be used widely for prostate chemoprevention. Therefore, bioactive food compounds such as epigallocatechin-3-gallate or resveratrol [4], [5], [6] represents an attractive alternative for prostate cancer chemoprevention, mainly due to their relatively low toxicity. Unfortunately, most of the previous studies have produced inconclusive results regarding their chemopreventive potential.

Recent identification of prostate cancer stem cells (CSCs) [7] has provided a new insight into prostate carcinogenesis. The ability of these cancer stem cells to self-renew and differentiate into bulk cancer cells suggested that they may be the origin of prostate cancer [7]. Moreover, the highly resistant nature of these CSCs to different chemotherapies suggested that CSCs may also contribute to treatment failure and disease relapse [8]. Interestingly, a number of bioactive food compounds have recently been shown to have anti-CSC effect. For example, we recently reported that gamma-tocotrienol extracted from palm oil inhibits prostasphere formation ability and tumorigenicity of prostate cancer cells [9], suggesting that gamma-tocotrienol is effective in suppressing prostate CSC properties. In addition, a triterpene extracted from fruits was also found to inhibit the self-renewal ability of liver CSCs and sensitize the liver tumor to cisplatin treatment [10]. These findings highlight the potential of bioactive food compounds as CSC targeting agent either for the prevention or for the treatment of prostate cancer.

Here, we demonstrated that the polysaccharopeptide (PSP) extracted from Turkey tail (known as *Coriolus versicolor* or Yun-zhi) targets prostate CSCs in vitro and suppresses tumor formation in vivo. Treatment of prostate cancer cell line PC-3 with PSP led to the down-regulation of CSC markers (CD133 and CD44) in a time and dose-dependent manner. Meanwhile, formation of prostasphere, a major property of prostate CSCs, was completely suppressed in PC-3 cells in the presence of PSP. Furthermore, PSP pre-treatment significantly suppressed tumor initiation of PC-3 cells in immunocompromised mice, suggesting that PSP suppresses the tumorigenicity of the PC-3 cells. More importantly, oral feeding of transgenic mice (TgMAP) that spontaneously develop prostate tumor with PSP was found to completely inhibit prostate tumor formation. Our findings support that PSP may be a potent chemopreventive agent against prostate cancer, possibly through targeting of the prostate CSC population.

## Results

### Effects of PSP on CSC marker expression

**Figure 1:**
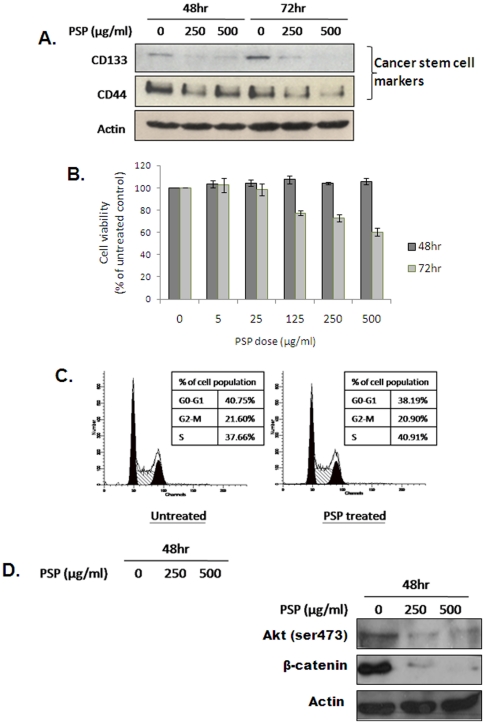
PSP down-regulates prostate CSC markers in PC-3 cells. A) Western blotting of prostate CSC markers CD44 and CD133 in PC-3 cells after PSP treatment. Note that PSP significantly down-regulates both stem cell markers in a dose- and time-dependent manner. B) Viability of PC-3 cells after treatment with 5, 25, 125, 250 and 500 µg/ml of PSP for 48 or 72 hr was measured with MTT assay. Results are presented as mean ± s.d. C) Flow cytometry analysis of PC-3 cells after treatment with 250 µg/ml of PSP for 72 hr. Note that no significant difference in cell cycle distribution was observed. D) Western blotting results for apoptotic markers (left panel) and stem cell maintenance proteins (right panel) in PC-3 cells after PSP treatment. Note that no changes in Bax and Bcl-2 or cleavage of PARP were detected.

PSP has previously shown to possess anti-cancer properties [11], [12], [13], although the underlying mechanisms are still unclear. To test if the anti-cancer effect of PSP is through targeting of CSC properties, we first investigated if PSP treatment affects the expression of prostate CSC markers in PC-3 cell line, which has been reported to contain CSCs. PC-3 cells were treated with 250 and 500 µg/ml of PSP for either 48 or 72 hr, and the expression of CSC markers such as CD133 or CD44 was examined by western blotting. As shown in Figure 1B, protein expression of CD133 was significantly down-regulated after PSP treatment in a time and dose-dependent manner. Downregulation of CD44 was also observed after PSP treatment, although the effect was less obvious. However, examination of the mRNA level of both CD133 and CD44 revealed that the downregulation of both proteins by PSP is not due to inhibition of gene transcription (data no shown). Nevertheless, these data suggest that PSP may be effective in targeting the putative prostate CSCs.

To test if the down-regulation of CSC marker expression by PSP is due to a decrease in cell viability, PC-3 cells treated with PSP at different dosages (0, 5, 25, 125, 250 and 500 µg/ml) for 24, 48 or 72 hr were examined by MTT assay. Interestingly, 48 hr of PSP treatment did not have any observable effects on cell viability, even though the same treatments was found to significantly suppress the expression of CSC markers (Figure 1B & 1C). Meanwhile, PSP treatment also failed to induce cell cycle arrest or apoptosis, as evidenced by the lack of sub-G1 population in the result of flow cytometry analysis (Figure 1D). This was further confirmed by examination of apoptosis-associated proteins (i.e. Bax, Bcl-2 and cleaved PARP) (Figure 1E). However, the Akt/β-catenin pathway, which is responsible for the enrichment of CSCs in breast cancer, was drastically inhibited by PSP treatment. As shown in Figure 1D, activation of AKT by phosphorylation at ser 473 was inhibited by PSP at both doses, which was accompanied by complete disappearance of β-catenin expression (Figure 1E).

### PSP inhibits prostasphere formation of prostate cancer cells under non-adherent culture conditions

**Figure 2:**
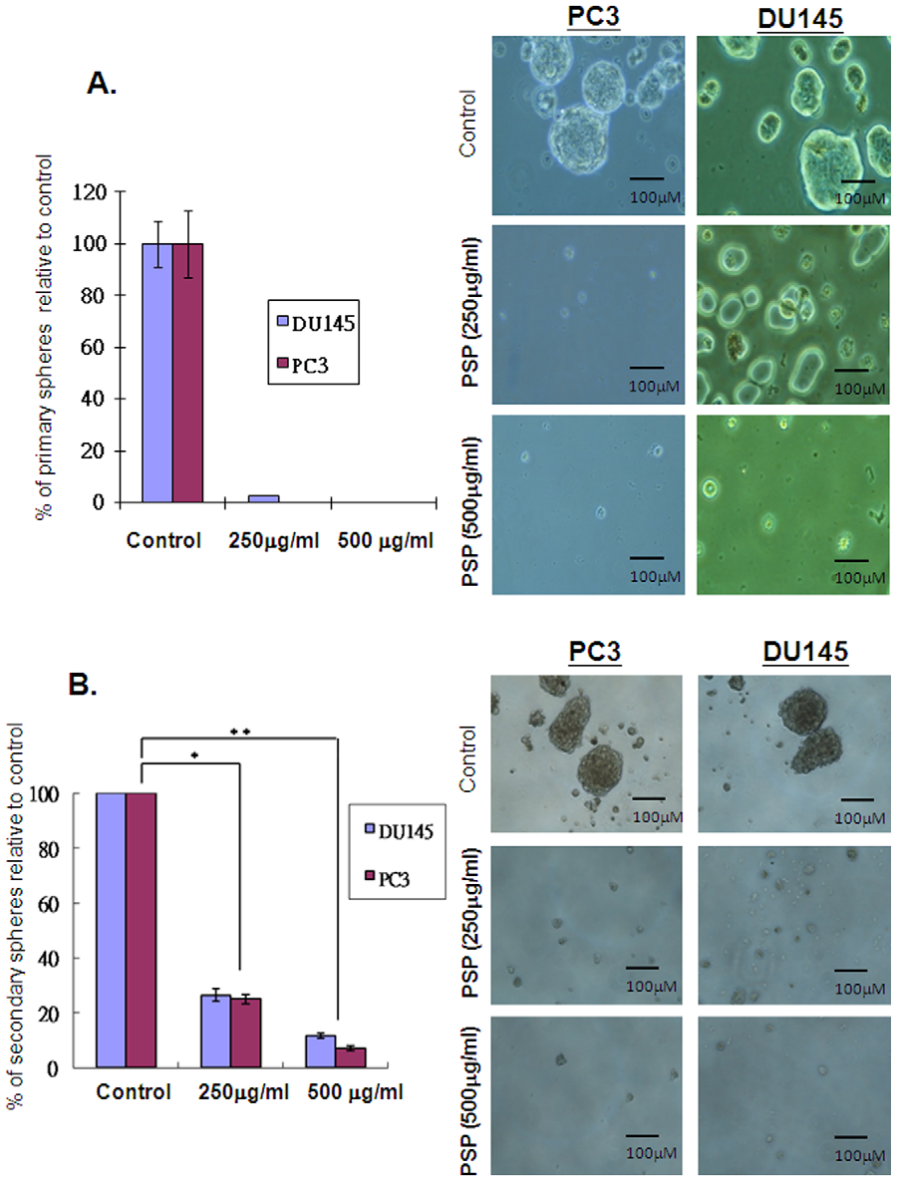
Effects of PSP on CSC properties. A) Spheroid formation assay was performed with PC-3 and Du145 cells. Two hundred of cells were seeded onto polyHEMA pre-coated plates and treated with either 500 µg/mL of PSP or vehicle for 14 days. The number of prostaspheres formed was counted, and the result was presented as the mean ± s.d. Note that γ-T3 treatment efficiently suppresses the spheroid formation ability of PC-3 cells. Image of the prostaspheres was captured under microscope. Note that no prostaspheres can be found in cells treated with 500 µg/mL of PSP. (B) PSP inhibited the formation of secondary prostaspheres. Primary prostaspheres were dissociated and re-seeded into polyHEMA pre-coated plate. PSP was added 24 hr after the plating. Note that prostasphere formation was inhibited by more than 70% and 90% in the presence of 250 µg/mL and 500 µg/mL of PSP respectively. * *P*<0.001, ** *P*<0.0001, *t* test.

The ability to form prostaspheres in non-adherent culture is one of the characteristics of prostate CSCs [14], [15], [16]. To confirm that PSP treatment can inhibit prostate CSC properties, prostasphere formation of PC-3 was studied in the presence or absence of PSP. As shown in Figure 2A, culturing of both PC-3 and DU145 cells for 14 days under non-adherent conditions results formation of prostaspheres, further confirming the presence of a stem-like population within both cell lines. Strikingly, addition of PSP into the medium drastically inhibited prostasphere formation in both cell lines. In particular, no prostaspheres was found in either cell line in the presence of 500 mg/ml of PSP, suggesting that PSP treatment significantly eliminated the prostate CSCs. To further proved that PSP is effective in inhibiting prostasphere formation, primary prostaspheres with enriched CSC population were dissociated and re-seeded into non-adherent culture condition to allow for the formation of secondary prostaspheres. Consistent with the result from the primary spheroid formation assay, PSP treatments significantly suppress the number of prostaspheres found in each cell line, although the higher dosage of PSP (500 mg/ml) was unable to completely eliminate all the secondary prostaspheres. Nonetheless, these results suggest that PSP is effective in suppressing the CSC properties of prostate cancer cells.

### PSP significantly reduces the tumorigenicity of prostate cancer cells *in vivo*

**Figure 3:**
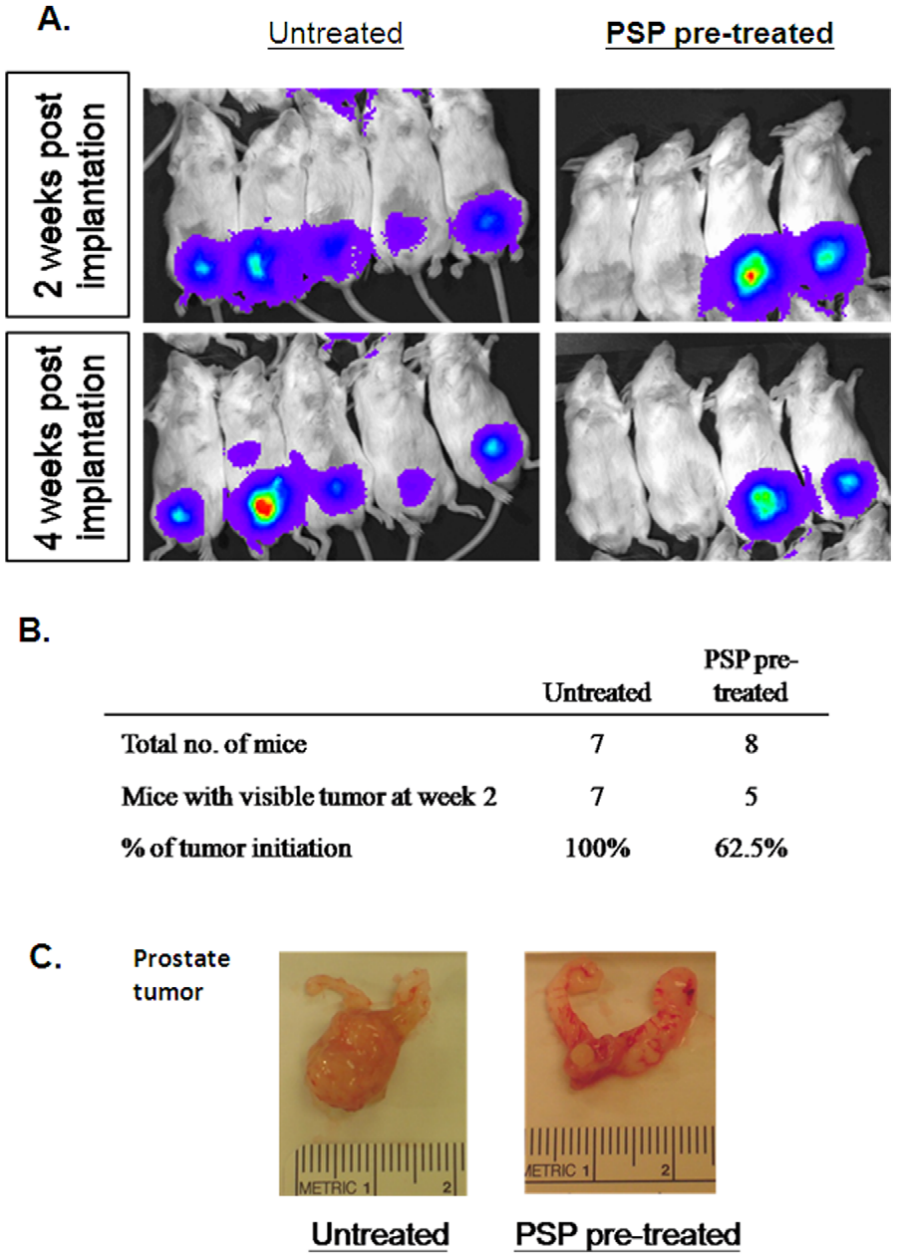
PSP inhibits tumorigenicity of PC-3 cells *in vivo*. A) Bioluminescent images of SCID mice orthotopically injected with PC-3-luc cells for two weeks. SCID mice in the upper row were injected with vehicle-treated PC-3-luc cells, whereas mice in the bottom row were injected with PSP-treated PC-3-luc cells. B) Table summarizes the percentages of mice developing detectable tumors at week 2. Approximately 40% of mice in the PSP pretreated group did not form detectable tumors, whereas 100% tumor formation was found in the control group (p = 0.07). C) Selected *ex vivo* images of the prostate from both groups. Note that in PSP-treated mice with negative luciferase signal, no visible tumor were found in the prostate tissue.

Since CSC is responsible for cancer initiation, it is possible that PSP treatment may inhibit the tumor formation ability of PC-3 cells in vivo. To test this hypothesis, we first treated PC-3 cells that stably expressing the luciferase protein (PC-3-Luc) with PSP for 72 hr before orthotopically injected them into the SCID mice. As examined by bioluminescence imaging, all of the mice that were injected with vehicle-pre-treated PC-3-luc cells formed tumors two weeks after the implantation (Figure 3A). Intriguingly, three out of the eight mice that were injected with PSP pre-treated PC-3-luc cells failed to develop tumors even at week four post implantation (Figure 3A&B). The lack of tumors in the PSP-pre-treated group was further confirmed by examination of the mouse prostate glands at the end of the experiment (Figure 3C). Taken together, our results suggested that PSP was effective in reducing the tumorigenic potential of prostate cancer cells, which is an essential characteristic of CSCs.

### Oral administration of PSP fails to inhibit prostatic intraepithelial neoplasia (PIN) development in TgMAP mice

**Figure 4:**
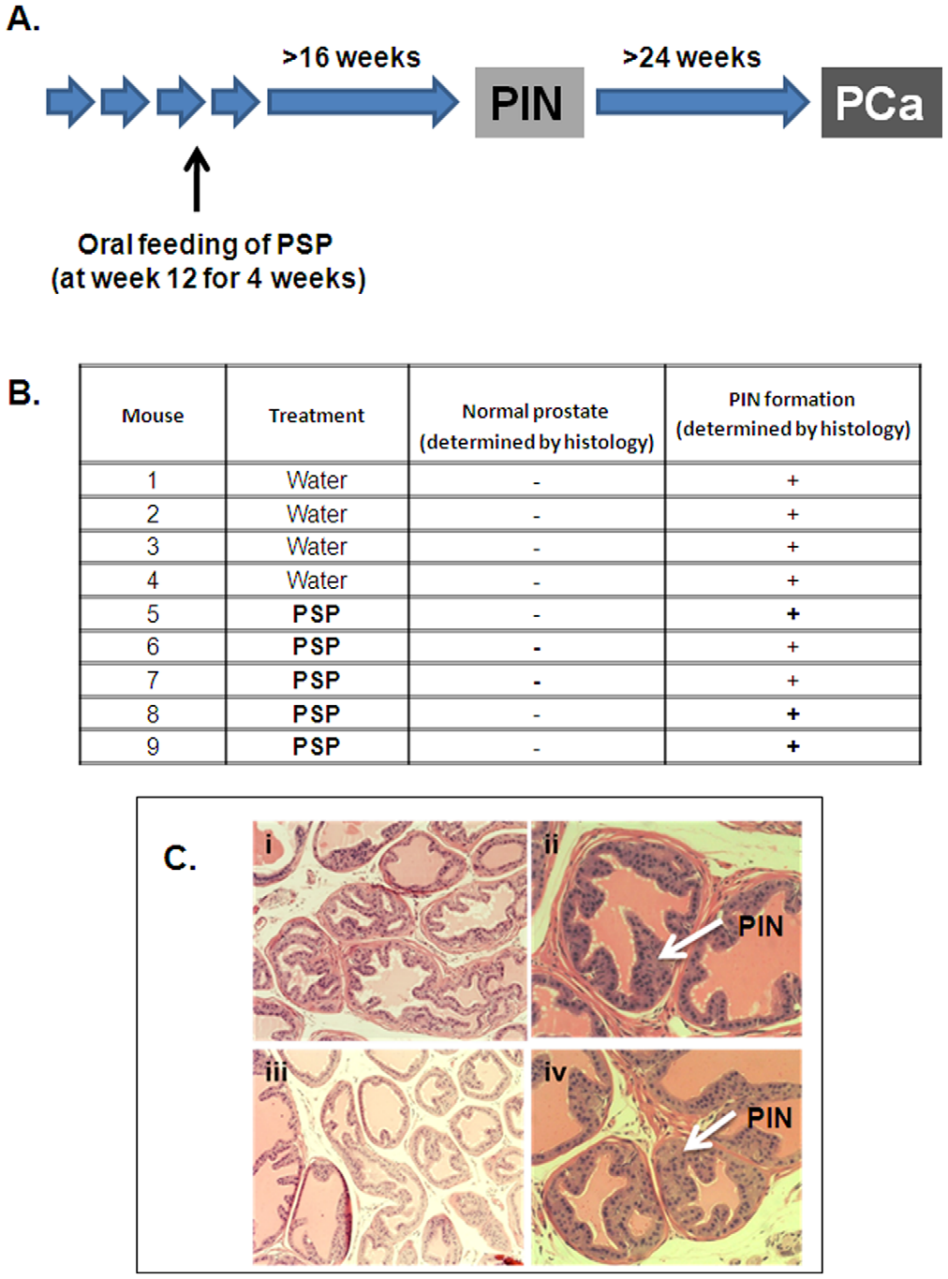
Effect of PSP on PIN development in the TgMAP transgenic mouse model. A) Time frame of the PIN and PCa development in TgMAP mice and the schedule of the PSP treatment. Fourteen-week old TgMAP mice were treated with 200 mg/kg of PSP by oral gavage feeding for 4 weeks and sacrificed at the time when PIN has developed (20 weeks old). The table summarizes the results of the histology examination of the prostate from the vehicle- and PSP-treated TgMAP mice. C) Representative photos of the Hematoxylin & Eosin staining of the prostatic tissues from the TgMAP mice. Note that both control- and PSP-treated TgMAP mice developed prostatic intraepithelial neoplasia (PIN), as indicated by the arrows.

The effect of PSP on prostate CSCs supports the hypothesis that it may have a chemopreventive effect against prostate cancer. To test this hypothesis, we have employed a transgenic mouse model that spontaneously develops adenocarcinoma of the prostate (TgMAP) [17], [18]. The TgMAP mice develop PIN between 16–20 weeks of age and progress to prostate adenocarcinoma after week 24 (Figure 4A). Because PIN is considered as the pre-malignant lesion and the most important risk factor of prostate cancer [19], we first tested if PSP administration affected PIN development in TgMAP mice. Five TgMAP mice (14-weeks old, at least 2 weeks before PIN development) were fed with 200 mg/kg of PSP for 4 weeks. Four mice of the same age were fed with water only for the same period of time. All mice were sacrificed at 20 weeks old and prostatic tissues were collected and sectioned for histology. As shown in Figure 4B&C, no differences were observed between the control and PSP-treated TgMAP mice regarding to the gross anatomy and the histology of the prostate gland. At low power magnification, tissue sections from both groups retained glandular structures, and at high power, PIN was detectable in both the control and PSP-treated mice. These results suggest that 4 weeks of PSP consumption was unable to stop the development of PIN.

### Prolonging PSP consumption inhibits prostate cancer development in TgMAP mice

**Figure 5:**
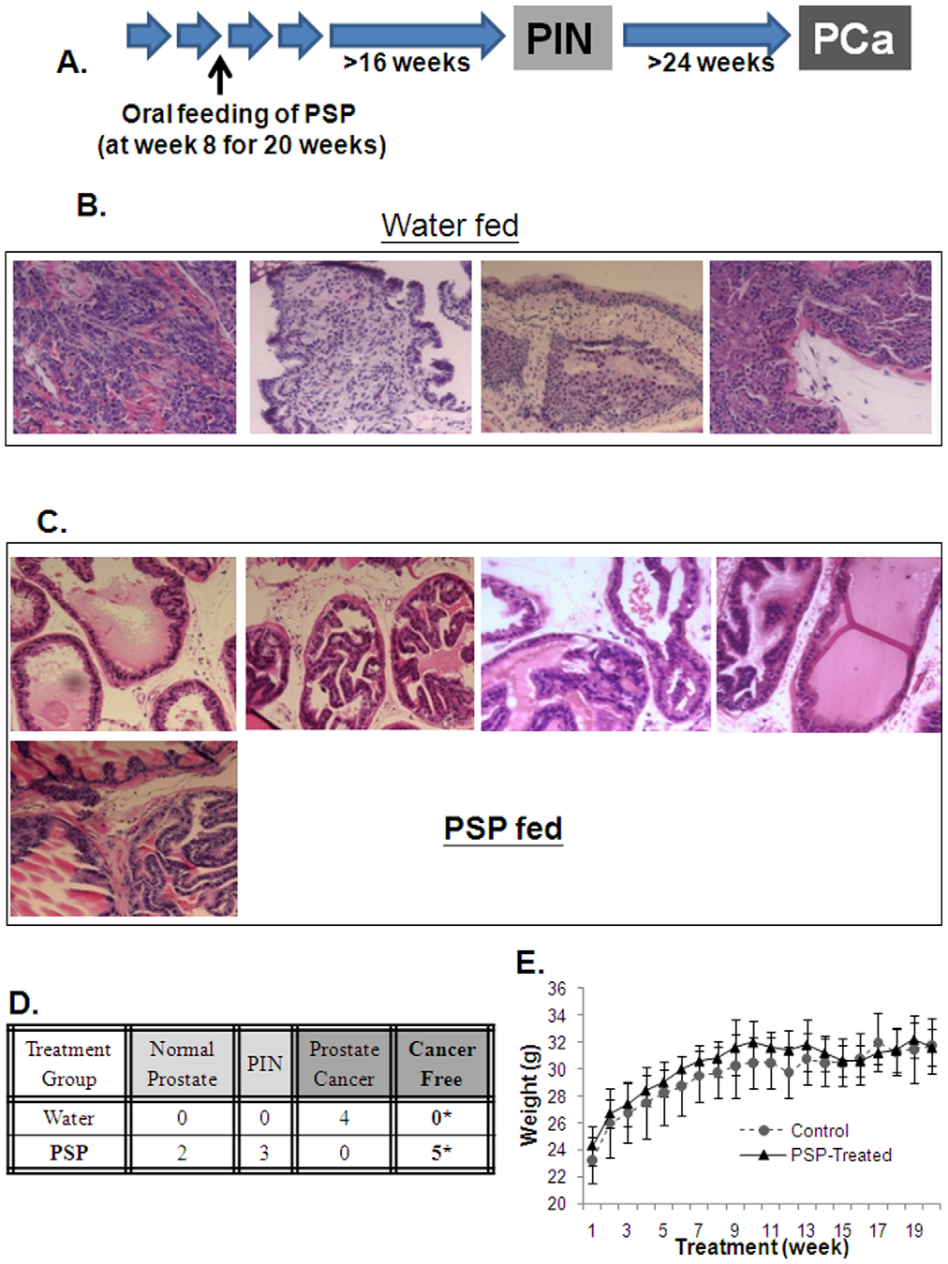
Effect of PSP on prostate tumor development of the TgMAP transgenic mouse model. A) Outline of the schedule for PSP treatment. Eight-week old TgMAP mice were treated with 300 mg/kg of PSP by oral gavage feeding for 20 weeks and sacrificed at age of 28 weeks. B & C) Representative photos of the Hematoxylin & Eosin staining of the prostate tissues from the vehicle and PSP-treated TgMAP mice. Note that tumors were found in all of the mice that were treated with vehicle only but were absent in all the PSP-treated mice. D) The table summarizes the results of the histology examination of the prostate tissues from the vehicle and PSP-treated TgMAP mice. *P<0.05 compared to control treatment by Fisher's exact test. E) Average body weight of the mice during the PSP treatment.

The failure to inhibit PIN formation by PSP treatment may due to insufficient dose or treatment length. We therefore tested if a higher dose and longer period of PSP consumption may affect prostate tumor formation using the same model. Five TgMAP mice (8-weeks old) were fed with 300 mg/kg of PSP for a total of 20 weeks (Figure 5A). Four mice at the same age were again fed with water only for the same period of time. All mice were sacrificed at 28 weeks old when prostate tumors were formed, with prostatic tissues collected and sectioned for histology. As shown in Figure 5B, tumors were found in different sections of the prostate gland from all of the mice that were fed with water only. Surprisingly, examination of all of the prostate section revealed that none of the mice that were fed with PSP bare any prostate tumors (Figure 5C), suggesting that PSP treatment completely inhibited prostate tumor formation in the TgMAP mice. Meanwhile, whereas three of the PSP-fed mice were found to have PIN, the other two mice were found to have completely normal prostates (Figure 5D). Furthermore, consistent with the low toxicity of PSP, long term consumption appears to have no side effect on the mice, as judged by the body weight changes and physical signs (data not shown) (Figure 5E). These findings strongly suggest that oral intake of PSP may be a safe and effective chemopreventive agent against prostate cancer.

## Discussion

PSP has previously been demonstrated to induce apoptosis and inhibit growth of a wide-range of cancer cells which includes breast [20], [21], [22], liver [23] and prostate cancer [24], although the mechanisms underlying its anti-cancer effects remain poorly understood. Here, we demonstrated for the first time that PSP has anti-CSC effects, as evidenced by the downregulation of CSC markers and the suppression of prostasphere and tumor formation.

Prostate CSCs were first identified by Collins et al. in 2005 using CD44+/alpha2beta1hi/CD133+ as the cell surface markers [25]. Using similar approaches, CSCs have also been identified in prostate cancer cell lines such as LNCaP [26], DU145 [26], [27] and PC-3 [28], [29]. These prostate CSCs not only express high level of CD133 and CD44, but are also highly tumorigenic when compared to the non-CSC population. The fact that PSP can significantly suppress the expression of both CD133 and CD44, as well as the tumorigenicity of PC-3 cells, clearly indicates the effectiveness of PSP in targeting prostate CSCs. As demonstrated by Hsieh et al [24], PSP is effective on induction of apoptosis and inhibition of cell proliferation in LNCaP cells. However, its effect were much less prominent in androgen independent prostate cancer cell lines such as PC-3. This is indeed consistent with our finding, which showed that PSP can suppress CSC properties without inducing any detectable cell cycle arrest or apoptosis. Nevertheless, the finding that both Akt phosphorylation and β-catenin expression were also down-regulated by PSP (Figure 1E) suggests that PSP may act by inactivating the Pten/Akt/β-catenin pathway to inhibit CSC renewal. This recently identified stem cell maintenance pathway was shown to play a key role in the regulation of prostate and mammary stem cell populations [16], [30]. Aberrant activation of the Akt/β-catenin pathway through the knockdown of Pten was found to enrich the mammary stem cell population, leading to the induction of hyperplastic lesions in the mouse [30]. Similarly, knockdown of Pten in prostate cancer cells was also found to enhance prostasphere formation ability and tumorigenicity of the cells [16]. Therefore, the the loss of “stemness” of prostate CSCs after PSP treatment may be due to down-regulation of the Pten/AKT/β-catenin pathway.

One of the key properties of stem cells is their ability to form spheres in non-adherent, serum-free conditions [31]. Indeed, spheroid formation assays have recently been used to identify and to enrich putative CSCs [16], [32], [33], [34]. Consistent with previous studies, both prostate cancer cell lines PC-3 and DU145 were able to form prostaspheres in non-adherent culture [16], suggesting the presence of CSCs within these cell lines. These primary prostaspheres, which are resistant to chemotherapeutic drugs [9], are highly sensitive to PSP treatment (Figure 2A). In addition, the secondary prostaspheres were significantly inhibited in a dose-dependent manner (Figure 2B), supporting that PSP is effective in eliminating prostate CSCs *in vitro*.

Prostate CSCs is believed to be origin of prostate tumor, which have the ability to self-renew and differentiate into the bulk tumor [35]. The fact that PSP pretreatment can significantly inhibit the tumorigenicity of PC-3 cells (Figure 3) not only highlights the anti-CSC effect of PSP, but also suggests that PSP may have chemopreventive effects against prostate cancer. We tested this hypothesis using a recently developed transgenic mouse model of prostate cancer (TgMAP) [17], [18]. The stepwise development of the prostate tumor (from low grade PIN to gross tumor) in the TgMAP mouse highly mimics the pathogenesis of human prostate cancer, although it may not totally reflect the complex nature of prostate carcinogenesis. Nonetheless, it allowed us to develop an optimal PSP treatment dosage and time frame. Whereas four weeks of PSP oral consumption at 200 mg/kg failed to produce any differences in PIN development, complete inhibition of prostate tumor formation was achieved after 20 weeks of oral PSP feeding at 300 mg/Kg. Meanwhile, the suppression of PIN formation by PSP further suggested that the chemopreventive effect of PSP may due to suppression of the tumor initiation at early stage. The extremely low toxicity and the highly potent anti-CSC effect of PSP warrants further evaluation of its chemopreventive effect in human clinical trials.

In summary, we have demonstrated, for the first time, that PSP treatment not only inhibits CSC properties, but also effectively suppresses prostate tumor formation. Our results suggest that PSP may be an effective agent for prostate cancer chemoprevention.

## Materials and Methods

### Polysaccharopeptide (PSP)

PSP extracted from Yun-zhi was kindly provided by Wonder Herb Health Products, Ltd. The PSP powder was dissolved in autoclaved Milli Q water at a concentration of 30 mg/ml by mixing in a rotator at 4°C overnight. The PSP solution was stored at 4°C. For cell culture study, PSP stock was sterilized with 0.2 µm filtration prior to use. In the animal study, PSP was fed directly to mice.

### Cell lines and culture conditions

Prostate cancer cell lines PC-3 and DU145 (ATCC, Rockville, MD) were maintained in RPMI 1640 medium (Invitrogen, Carlsbad, CA) supplemented with 1% (w/v) penicillin-streptomycin (Invitrogen, Carlsbad, CA) and 5% fetal bovine serum (Invitrogen, Carlsbad, CA). All cell lines were kept at 37°C in a 5% CO_2_ environment. Luciferase-expressing PC-3 cell line was generated in our previous study.

### TgMAP prostate tumor model

TgMAP C57/BL6 mice at week 8 and 14 were administered with PSP at 200 mg/kg for 4 weeks (n = 5) or 300 mg/Kg for 20 weeks (n = 5) respectively by oral gavage feeding (5 days per week). Control group were fed with water only for the same period of time. Mice were sacrificed (at the age of 20 weeks for 200 mg/Kg treatment group and 28 weeks for the 300 mg/Kg treatment group) and prostate tissues were collected, fixed in 10% formalin and embedded in paraffin. The whole prostate was cut into 4 µm sections and one in every five consecutive sections was stained with H&E. Histology examination was performed by Dr. K.W. Chan (pathologist, HKU). Statistical difference was determined by Fisher's exact test and was considered as significant if p<0.05. Animal ethics was approved by the Committee on the Use of Live Animals for Teaching and Research (CULATR) with the approval no. of 1694-08. All animal handling procedures were carried out according to the guidelines of the Committee on the Use of Live Animals in Teaching and Research (CULATR), The University of Hong Kong.

### Spheroid formation assay

The spheroid formation assay was modified from a previously reported protocol [36]. Briefly, PCa cells (200 cells per line) were seeded onto 12-well polyHEMA (Sigma)-coated plates. Cells were grown in DMEM/F12 medium (Invitrogen, Carlsbad, CA) for 14 days supplemented with 4 µg/mL insulin (Sigma), B27 (Invitrogen), 20 ng/mL EGF (Sigma), and 20 ng/mL basic FGF (Invitrogen) with PSP at either 250 µg/mL and 500 µg/mL. For serial passage of primary spheres, the primary spheres were treated with PSP for the above doses for 72 h and subsequently collected, dissociated with trypsin, and resuspended in DMEM/F12 medium with the above supplements. Each experiment was repeated in triplicate, and each data point represents the mean and standard derivation. Statistical difference was determined by Student's *t*-test and was considered as significant if p<0.05.

### Cell viability assay

Cell viability upon PSP treatment was measured by a 3-(4,5-Dimethyl thiazol-2-yl)-2,5-diphenyl tetrazolium bromide (MTT) assay as described previously [37]. Briefly, cells were seeded on 96-well plates and treated with different concentrations of PSP for the indicated time. At the end of the treatment, MTT (Sigma, St. Louis, MO) was added to each well, and wells were incubated for 4 hr at RT. DMSO was then added to each well to dissolve the formazan crystals. The plate was incubated for a further 5 min at RT, and the optical density (OD) was measured at a wavelength of 570 nm on a Labsystem multiscan microplate reader (Merck Eurolab, Dietikon, Switzerland). All individual wells were analyzed in triplicate. The percentage of cell viability was presented as the OD ratio between the treated and untreated cells at the indicated concentrations.

### Western Blotting

Detailed experimental procedures have been described previously [37]. Briefly, cells were lysed with RIPA buffer (50 mM Tris-HCl pH 8.0, 150 mM NaCl, 0.5% deoxycholic acid, 1% NP-40, 0.1% SDS) with protease inhibitors (1 mg/ml aprotinin, 1 mg/ml leupeptin, 1 mM PMSF) and the protein concentrations were determined using a D_C_ Protein Assay Kit (Bio-Rad, Hercules, CA). Proteins were resolved in SDS- polyacrylamide gel by electrophoresis and then transferred onto Hybond-P PVDF membrane (Amersham Biosciences, Piscataway, NJ). The membranes were blocked by 10% non-fat dry milk in TBS-T or 3% non-fat dry milk in TBS and incubated with primary antibodies at room temperature against Akt (ser 473), Bcl-2, PARP (Cell signaling, Technology Inc, Beverly, MA), CD133 (Miltenyi Biotec, Auburn, CA), CD44, β-catenin and β-actin (Santa Cruz Biotechnology, Santa Cruz, CA) for 1 hr at room temperature. After washing with TBS-T, the membrane was incubated with either anti-mouse or anti-rabbit IgG secondary antibodies, and the signals were visualized using the ECL plus western blotting system (Amersham, Piscataway, NJ).

### Cell cycle analysis

Cells were fixed with 1 ml ice cold 70% ethanol at 4°C. After fixation, cell pellets were collected by centrifugation, resuspended with 500 µl PBS, and then incubated at 4°C a day before performing flow cytometry. On the next day, cells were stained with propidium iodide (50 µg/ml) and RNase (1 µg/ml) for 30 min. Cell cycle analysis was performed on a flow cytometer EPICS profile analyzer and analyzed using the ModFit LT2.0 software (Coulter, Miami, FL).

### Orthotopic implantation of PC-3-luc cells

The orthotopic model was established with procedures described previously [38]. Briefly, eight-week-old CB-17 SCID mice were anesthetized and placed under a dissecting microscope. An incision at the midline of the abdomen was made, exposing the dorsal prostate at the base of the bladder. Equal amounts of viable PC3-luc cells (2.5×10^4^) with or without prior PSP treatment were injected into the dorsal prostates of the mice. The organs were replaced, and the abdomen was closed. Tumor development was monitored by measuring the bioluminescent signal every two weeks for six weeks after tumor implantation. Mice were sacrificed at the end of the experiment and prostate tissues were collected for physical examination. Statistical difference was determined by a two-tailed *t*-test and was considered significant if p<0.05. All surgical and animal handling procedures were carried out according to the guidelines of the Committee on the Use of Live Animals in Teaching and Research (CULATR), The University of Hong Kong.
