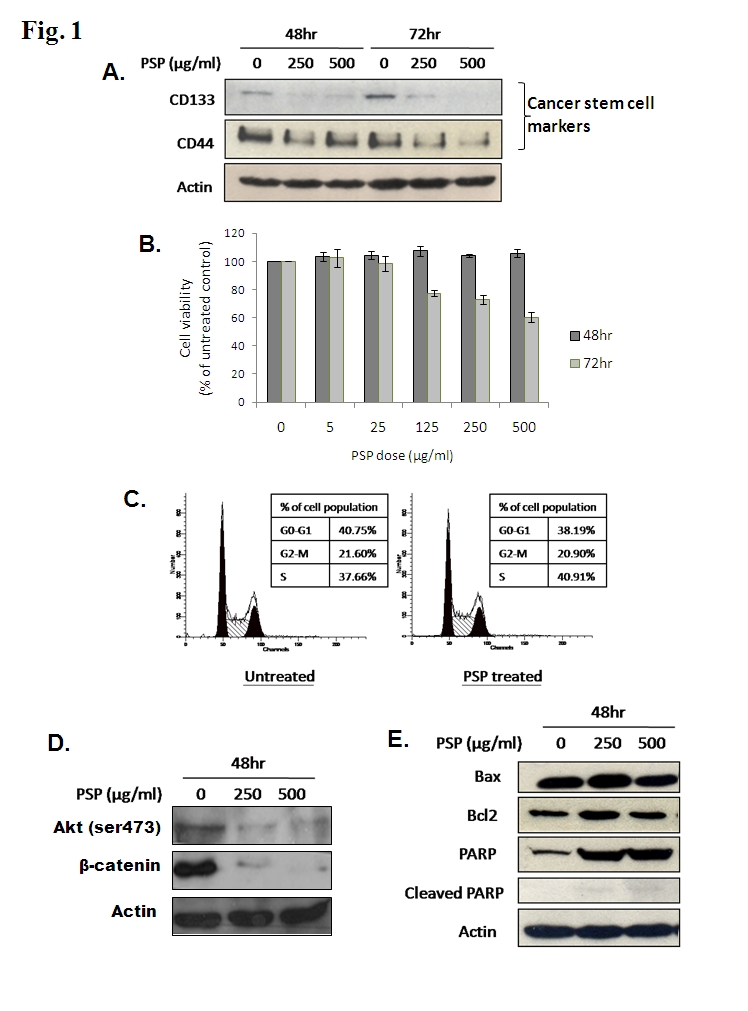# Correction: Chemopreventive Effect of PSP Through Targeting of Prostate Cancer Stem Cell-Like Population

**DOI:** 10.1371/annotation/0f6309be-936c-4974-97bf-ed3a98289cd9

**Published:** 2011-06-02

**Authors:** Sze-Ue Luk, Terence Kin-Wah Lee, Ji Liu, Davy Tak-Wing Lee, Yung-Tuen Chiu, Stephanie Ma, Irene Oi-Lin Ng, Yong-Chuan Wong, Franky Leung Chan, Ming-Tat Ling

A panel in Figure 1 is missing. The correct Figure 1 can be viewed here: 

**Figure:**