# Correction: Chemopreventive Effect of PSP Through Targeting of Prostate Cancer Stem Cell-Like Population

**DOI:** 10.1371/annotation/b0312c4c-e06a-47ef-9e9c-b044dbfa3d6a

**Published:** 2011-06-02

**Authors:** Sze-Ue Luk, Terence Kin-Wah Lee, Ji Liu, Davy Tak-Wing Lee, Yung-Tuen Chiu, Stephanie Ma, Irene Oi-Lin Ng, Yong-Chuan Wong, Franky Leung Chan, Ming-Tat Ling

There are multiple errors in the legends of Figure 1 and Figure 2. The correct legends are:

Figure 1. PSP down-regulates prostate CSC markers in PC-3 cells.

(A) Western blotting of prostate CSC markers CD44 and CD133 in PC-3 cells after PSP treatment. Note that PSP significantly down-regulates both stem cell markers in a dose- and time-dependent manner. (B) Viability of PC-3 cells after treatment with 5, 25, 125, 250, and 500 μg/ml of PSP for 48 or 72 hr was measured with MTT assay. Results are presented as mean ± s.d. (C) Flow cytometry analysis of PC-3 cells after treatment with 250 μg/ml of PSP for 72 hr. Note that no significant difference in cell cycle distribution was observed. (D and E) Western blotting results for stem cell maintenance proteins and apoptotic markers in PC-3 cells after PSP treatment. Note that no changes in Bax and Bcl-2 or cleavage of PARP were detected.

Figure 2. Effects of PSP on CSC properties.

(A) Spheroid formation assay was performed with PC-3 and Du145 cells. Two hundred of cells were seeded onto polyHEMA pre-coated plates and treated with either 500 mg/mL of PSP or vehicle for 14 days. The number of prostaspheres formed was counted, and the result was presented as the mean ± s.d. Note that PSP treatment efficiently suppresses the spheroid formation ability of PC-3 cells. Image of the prostaspheres was captured under microscope. Note that no prostaspheres can be found in cells treated with 500 mg/mL of PSP. (B) PSP inhibited the formation of secondary prostaspheres. Primary prostaspheres were dissociated and re-seeded into polyHEMA pre-coated plate. PSP was added 24 hr after the plating. Note that prostasphere formation was inhibited by more than 70% and 90% in the presence of 250 mg/mL and 500 mg/mL of PSP respectively . *P<0.001, **P<0.0001, t test. 

